# Book Review

**Published:** 1915-06

**Authors:** 


**Outlines of Zoology.** By J. Arthur Thomson, M.A., LL.D. Sixth Edition. Pp. xxi. 885. London: Henry Frowde and Hodder & Stoughton. 1914. Price 12s. 6d.—A new edition of this text-book is always a treat for students of zoology and biology, as it contains in the most readable way all the facts required by the student of zoology, whose outlook is bounded purely and simply by anatomical and developmental facts. It is, moreover, greatly valuable for the student of natural history, as it contains packed away in an unostentatious manner numerous facts connected with the why and the wherefore of animal life. The present edition shows considerable advance on its predecessors in the number of facts dealt with, and also in the new types studied, and especially in the new diagrams, which will be a veritable treat to a large number of students. We congratulate the author on being able, amidst all his other work, to publish such an up-to-date manual, and also on the fact that, in setting things plainly, lucidly, and attractively before his readers, his hand has not lost its cunning.

**The Newer Physiology in Surgical and General Practice.** By A. Rendle Short, M.D., B.S., B.Sc. Third Edition. Pp. 256. Bristol: John Wright & Sons Ltd. 1914. Price 5s. net.—Within a period of less than three years Dr. Rendle Short has had to bring out three editions of his work. This proves the necessity for a book dealing with the subjects comprised in it. So many advances have been made in our knowledge since the first edition, that the third now before us has had to be very largely re-written. Hence the alteration in the title from the "New" to the "Newer" Physiology. Entirely new chapters are those on "Vitamines," the "Genital Glands," and "Surgical Shock." The latter embodies Dr. Short's Hunterian Lecture before the Royal College of Surgeons. There are new paragraphs on the dangers of the adrenalin-chloroform combination, and on the functional relations of the ileo-cæcal valve and movements of the stomach. Important additions are made to the chapters on the "Thyroid Gland" and "Physiology of the Spinal Cord," and there are others of perhaps less importance. It is to be hoped that if there be anyone who has escaped reading this little book he will get it without delay, as it cannot fail to be both interesting and instructive to him.

**Essentials of Physiology.** By F. A. Bainbridge, M.A., M.D., and J. Acworth Menzies, M.D. Pp. viii. 434. London: Longmans, Green & Co. 1914. Price 10s. 6d. This is yet another new text-book of physiology to add to the two American and four English already in common use. It represents the teaching of the Durham school. The main feature is that here we have the briefest (416 pages) of the modern text-books that profess to be adequate for the medical student, nor are the facts set forth in so synoptic a fashion as to rob them of interest. The illustrations are numerous and well-chosen. The authors have displayed a good deal of judgment in their choice of what to include and what to eliminate in the text, and the book is one of the best, if not the best, for students who require no more physiology than they will need to get through a pass examination without overburdening their memories. There are half-a-dozen statements scattered here and there with which your reviewer does not happen to agree (uselessness of dextrose enemata, influence of nervous system on secretion of milk. origin of cholesterin in bile from the blood instead of from gal bladder, nature of secretion of lymph, reducing division ascribed to the first instead of the second formation of polar bodies), but some of these are matters of opinion, and none of them of great importance.

**Essentials of Human Physiology.** By D. Noël Paton, M.D. Fourth Edition. Pp. xxiii, 557. London: Longmans, Green and Co. 1914. Price 12s.—A book which has already reached a fourth edition is obviously fulfilling a useful mission. The present volume is brought well up to date. Amongst the more interesting of the new paragraphs may be mentioned Wilson's work on the x-chromosomes, always present in the ovum, but only present in half the spermatozoa. Some embryos, therefore.

start life with two x-chromosomes and become female, whereas others start with only one, and become male. Sex would, therefore, appear to be a matter of luck, which spermatozoon will chance to get there first. Noel Paton's text-book is decidedly smaller than most of its rivals, although it contains a great bulk of facts; it is therefore rather synoptical in parts. The diagrams are of the simplest possible description. Those who find themselves better able to read and appreciate brief, unadorned dogmatic statements than a more detailed, perchance a more interesting, discussion, will find this book very much to their mind.

**Elements of Surgical Diagnosis.** By Sir A. Pearce Gould, K.C.V.O., M.S., F.R.C.S. Fourth Edition. Revised and enlarged by the Author, with the assistance of Eric Pearce Gould, M.A., M.Ch., F.R.C.S. Pp. xiv. 723. London: Cassell and Company Ltd. 1914. Price 10s. 6d.—After a lapse of eleven years a new edition of this well-known manual has been brought out by the distinguished author, assisted by his son. The book thus revised is thoroughly up to date; for instance, it gives skiagrams illustrative of pyelography for various affections of the kidney. It is full, yet simple, concise and eminently practical; it confines itself strictly to the point, which is the utilisation of symptoms, physical signs and special modes of examination in order to make a diagnosis in surgical cases. Although we have perused it with critical care, we did not observe any omissions of importance, except that we think the sigmoidoscope ought to be mentioned in the chapter on diseases of the rectum.

**The Operative Treatment of Fractures.** By Sir W. Arbuthnot Lane, Bart., M.S., F.R.C.S. Second Edition. Pp. 184. London: The Medical Publishing Company Limited. 1914. Price 10s.—The author prefaces his remark by a brief statement of investigations made by him on the changes which the skeleton undergoes from the habitual assumption of attitudes of activity or of rest. These called his attention to the unsatisfactory treatment of fractures with regard to functional results. He illustrates by skiagrams the effectual attempts by Nature, following fracture of long bones in children, to restore the injured bone to its normal form by a process of absorption and deposit of bone, whereby an accurate alignment is obtained. On this he bases his arguments for the operative accurate replacement of fractures, in which even slight deformity exists, uncorrected, not even the clavicle would escape him, and age, he maintains, is no bar. His plate, screws and staples are the mechanical means he employs, the wire is to be shunned almost entirely. The staple he advises in fractures near joints where plate and screw are inapplicable.

The technique is dealt with in detail as of the utmost importance, and rightly so, but one feels that there are others also capable of carrying out such surgery aseptically. With compound fractures he sometimes uses the plate with drainage, but prefers to await perfect healing before its application. No mention is made of after-troubles in the form of pain due directly to the plate, or of sinuses appearing long after an apparently perfect primary union has taken place, necessitating its removal. Other methods, such as fixation by bone or ivory pegs are mentioned, but to be completely condemned, in what appears to be unwarrantable haste, in view of recent work. The book is worthy of perusal as to the results of the author's method which has proved of great value, and is a considerable advance in the treatment of certain fractures, but the ideal fixature is yet to be found.

**On Means for the Prolongation of Life.** By Sir HERMANN WEBER, M.D. Fourth Edition. Pp x. 235. London: John Bale, Sons & Danielsson, Ltd. 1914. Price 4s. 6d.—No one can be better qualified than the author to speak with authority on this subject, for he says: "Now in my ninety-first year I possess good health and a fair amount of strength, and am able, to some degree, to watch the progress in my old profession, in science and art and social matters, and I still enjoy the beauties of Nature in my country walks, and above all things intercourse with my children and their families and old friends." The book tells us how it has been done, but no matter what our personal aspirations may be—

"There 's many a slip 'twixt cup and lip."

The principal points emphasised by the author are summed up tersely in Shakespearean phraseology:—

"Though I look old, yet I am strong and lusty;
For in my youth I never did apply
Hot and rebellious spirits in my blood,
Nor did not with unbashful forehead woo
The means of weakness and debility,
Therefore my age is as a lusty winter,
Frosty, but kindly."

**The Students' Pocket Prescriber.** By H. A. HUSBAND, M.B., C.M. Fourth Edition. Pp. 152. Edinburgh: E. & S. Livingstone. 1914. Price 1s. 6d. net.—Contains directions for prescription writing, 487 prescriptions arranged according to the diseases they are intended to cure, the doses being given in imperial and metrical measures, and an appendix of miscellaneous information. As useful as most little books of this class.

**The Examination of the Urine.** By A. F. HEWAT, M.B., Ch.B. Fifth Edition. Pp. 212. Edinburgh: E. & S. Livingstone. 1914. Price 1s. 6d. net. Contains descriptions of the methods of examination of urine, blood, sputum, gastric contents and fæces. It is illustrated and carefully printed, and the methods seem to have been collected from standard works.

**The Biology of the Blood-Cells.** By O. C. Gruner, M.D. Pp. xii. 392. Bristol: John Wright & Sons Ltd. 1913. Price 21s. net.—This is not a text-book of hæmatology, but an elaborate study of the origin and development of the various types of cell found in the blood, with special references to the various problems which arise in the clinical study of diseased conditions. The literature has been very fully dealt with, as the long index of authors testifies; and the minute structure of the cellular elements has been studied with an elaboration which is almost bewildering. There is an index of hæmatological terms occupying thirty-three pages, which is of considerable value, and it not only attempts to co-ordinate the various terms used by individual workers, but also defines a number of theories or views in a succinct manner. Thus we find that there are ten synonyms for myeloma, and no less than twenty-four for leucoblast. The book is very fully and beautifully illustrated by photographs and colour prints. It should prove valuable to specialists and advanced students interested in this field of pathology.

**The Child's Diet.** By J. Sadler Curgenven, M.R.C.S. Second Edition. Pp. viii. 115. London: H. K. Lewis. 1914. Price 2s. 6d.—This little book is based on the actual experience of twenty years' general practice. The author gives one chapter to the feeding of infants, the remainder of the book deals with the feeding of older children. The advice given is generally sound, and the recipes at the end of the book will often be found of great assistance to the reader. We do not, however, agree that milk sugar is the best sugar for the bottle-fed infant, in our own experience refined cane is far better. The word "sterilisation" is used rather loosely, "pasteurisation" would more accurately describe the process the author recommends. With these few exceptions the teaching is practical and up to date, and we feel the book will thoroughly serve the purpose for which it has been written.

**The "Wellcome" Photographic Exposure Record and Diary, 1915.** London: Burroughs, Wellcome & Co. Pp. 280. Price 1s.—This useful little book is a veritable encyclopædia of photography, and gives clear and concise directions for every process. Exposure factors are given for all British and American plates and films, and the ingenious "Wellcome" Exposure Calculator gives the correct exposure for any plate or film at any time of the day or year. The inexperienced amateur will find all the information he seeks, and those more expert will find much that is helpful and suggestive.

**The Minor Horrors of War.** By A. E. Shipley, Sc.D. Pp. xvii. 166. London: Smith, Elder & Co. 1915. Price 1s. 6d. in paper cover, or 2s. in cloth.—Dr. Shipley has published as a small book his excellent articles which appeared in the *British Medical Journal* during the autumn and winter. The combination of scientific accuracy with a racy style is a refreshing novelty, and enables us to read of these lousy insects of all sorts without arousing the usual desire to scratch. To a sensitive reviewer this quality in such a book exhausts all ordinary terms of praise. The illustrations are works of art, and the type is admirable.

**Mechano-Therapeutics in General Practice.** By G. de Swietochowski, M.D. Pp. xiv. 141. London: H. K. Lewis. 1914. Price 4s. net.—This is a useful little book written specially for medical men. The author advocates the early use of massage and movements in the treatment of sprains, dislocations and fractures, and gives a clear description of the procedure to be followed in each form of injury. A few simple exercises for the treatment of scoliosis are given. The latter part of the book deals with disorders of the circulatory and respiratory organs suitable for treatment by massage and exercises. The writer points out the advantage, both to themselves and their patients, of medical men acquiring some knowledge of mechano-therapeutics, so as not to leave the treatment entirely in the hands of the more or less ignorant: "more medicine in massage, and more massage in medicine."

**Text-Book of Massage.** By L. L. Despard. Second Edition. Pp. xix. 413. London: Henry Frowde and Hodder and Stoughton. 1914. Price 12s. 6d. net.—In addition to the valuable subject-matter compiled in the first edition, new chapters on medical exercises and electricity have been added. The book should prove very useful to nurses and others intending to obtain a massage certificate.

